# Highly Sustainable Dyes Adsorption in Wastewater Using Textile Filters Fabricated by UV Irradiation

**DOI:** 10.3390/polym16010015

**Published:** 2023-12-19

**Authors:** Sujin Ryu, Young Ki Park, Jaeyun Shim, Seungju Lim, Minsuk Kim

**Affiliations:** 1 Advanced Textile R&D Department, Research Institute of Convergence Technology, Korea Institute of Industrial Technology (KITECH), 143 Hanggaulro, Ansan 15588, Republic of Korea; sjryu@kitech.re.kr (S.R.); parkyk@kitech.re.kr (Y.K.P.); sjaeyun@kitech.re.kr (J.S.); 2 Department of Fiber System Engineering, Dankook University, Yongin 16890, Republic of Korea

**Keywords:** dye adsorption, UV-photografted filter, dye adsorption filter, dye wastewater treatment, dye removal, dye removal filter

## Abstract

Vast amounts of dyeing wastewater released from the textile industry can not only cause water pollution but also have negative effects on the human body, such as skin irritation and respiratory diseases. Dye adsorption technology is necessary for the treatment of wastewater discharged from the dyeing industry and for environmental improvement. However, to remove dyeing wastewater, more energy and solvents are used to fabricate adsorbents, or excessive energy is used to filter dyeing wastewater out, resulting in more environmental pollution. Therefore, it is necessary to develop a method of filtering dyeing wastewater in a more environmentally friendly manner by minimizing the use of solvents and energy. In this study, we modified the surface of a textile substrate through UV irradiation to create a monomer capable of facilely bonding with dyes. Employing the UV photografting method, we were able to produce a dye adsorption filter in a more environmentally friendly manner, minimizing solvent usage and heat energy consumption required for absorbent synthesis. At a monomer concentration of 10%, the fabricated filter exhibited a dye removal efficiency of 97.34% after 24 h, all without the need for a pressure treatment or temperature increase. Moreover, it displayed an adsorption capacity of approximately 77.88 mg per 1 g of filter material.

## 1. Introduction

Currently, water pollution is a paramount global issue stemming from the improper discharge of industrial water and excessive use of chemical fertilizers in agriculture, road construction, building construction, and related activities [1]. Environmental preservation endeavors have become increasingly crucial, with a significant focus on addressing water pollution, particularly the discharge of dyeing wastewater from industries such as textiles, printing, dyeing, and food processing. Notably, the textile industry stands out as the largest contributor to dye wastewater, accounting for a substantial two-thirds of the total dye waste production [2,3]. Typically, a substantial portion, up to 80%, of dye molecules in a dye solution can be adsorbed by a substrate [4]. However, fabrics possess limited absorption capacities, capable of adsorbing only 25% of the dye solution [5]. Consequently, the remaining 75% or more of the dye ends up being discharged into wastewater [6,7]. Despite the direct health implications of discharged dyeing wastewater, including skin irritation, respiratory diseases, and potential carcinogenic and mutagenic effects, the annual production of dyes exceeds 200,000 tons, with over 150,000 tons being released into the environment [8,9]. In particular, Congo Red, an acidic dye, is used in various industries, such as textiles, paper, and printing, but is reported to be carcinogenic and toxic [10]. To tackle this issue, various international organizations and national agencies have established wastewater discharge standards, and research efforts are underway to eliminate dyes from discharged dyeing wastewater.

Presently, methods for removing dyes from dyeing wastewater encompass adsorption, coagulation, flocculation, ion exchange, irradiation, membrane filtration, nano-filtration, and ultra-filtration [10]. Adsorption is hindered by adsorbent costs, while coagulation and flocculation are ineffective against certain dye types, including acidic, azo, basic, and reactive dyes. Ion exchange is limited to specific dyes, and irradiation necessitates substantial dissolved oxygen, resulting in high expenses. Membrane or nano-filtration can be costly to manufacture, energy-intensive, and require frequent filter replacement due to pore clogging, necessitating pressure for filtration [11,12,13]. Of these methods, adsorption is the most widely used industrially, with ongoing research into various dye adsorbents, including activated carbon [14], lignin-based products [15,16,17], chitosan [18,19], cellulose-based beads [20], Metal–Organic Framework (MOF) [21,22,23,24], and other thermopolymerized composites [25,26].

Nonetheless, conventional dye adsorbents generate sludge upon dye adsorption, demanding centrifugation, expensive adsorbents, and excessive energy consumption during polymerization. Therefore, manufacturing a filter-type adsorbent offers the advantage of easy removal without centrifugation following dye adsorption. To effectively remove acidic and reactive dyes with sulfonic acid groups found in dyeing wastewater, a surface modification is essential to facilitate stronger bonds between the dyes and molecules. UV photografting is a surface modification method that consumes less energy than conventional thermal polymerization. It achieves this by using UV irradiation to extract hydrogen from the fiber surface, forming radicals that react with monomers to promote covalent bonding between the fiber filter and dye [27]. UV photografting operates at low temperatures, ensuring rapid polymerization initiation, preventing fabric damage, and facilitating fast drying, all while minimizing the release of harmful volatile organic compounds [28,29,30]. Additionally, UV photografting boasts low energy consumption and low-liquid-ratio polymerization, enabling the production of eco-friendly dye adsorption filters [30,31].

The primary aim of this study is to develop eco-friendly textile-based filters for dye removal of toxic Congo Red, widely used in various fields. The filter manufactured by UV photografting proposed in this study does not require heating, stirring, or pH adjustment during the adsorption process, and the dye can be removed simply by picking up the filter after adsorption, so even the adsorption process is environmentally friendly. Consequently, our research endeavors encompass the creation of filters optimized for easy dye removal through surface modification using UV photografting. We also intend to characterize these filters and evaluate their dye removal capabilities.

## 2. Materials and Methods

### 2.1. Materials

The nylon fabric used as the substrate for the textile filter was supplied by HYOSUNG TNC (Seoul, Republic of Korea); its characteristics are shown in Table 1.

The following chemicals were procured for the study: N-[3-(dimethylamino)propyl] methacrylamide (DMAPMAm) at a purity of 97% as a monomer for UV photografting, acetone with a purity of 98%, and Congo Red for solution of dye removal at 98%. These were obtained from SigmaAldrich in Seoul, Republic of Korea. Additionally, 2,4-Diethyl-9H-thioxanthen-9-one (DETX), with a purity of 98%, was purchased from Tokyo Chemical Industry Co. Ltd. in Chuo-ku, Tokyo, Japan. All chemicals and reagents were utilized as received and underwent no further purification.

### 2.2. Fabrication of the UV-Photografted Filters

**Figure 1:**
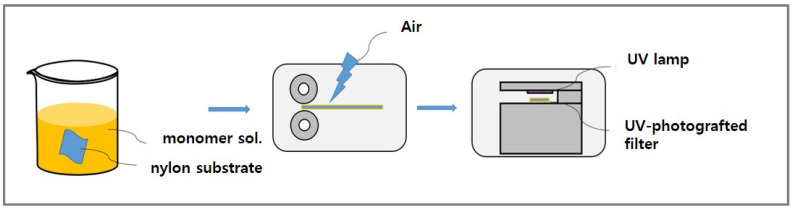
Process for the fabrication of dye adsorption filters by UV irradiation.

A schematic representing the UV irradiation process is depicted in Figure 1. To create a graft solution, 10 wt% of DMAPMAm, a monomer, was dissolved in acetone, along with Photoinitiator (DETX, 10% by weight). Subsequently, the nylon fabric was immersed in this graft solution, and a padder (Daelim Starlet Co., LTD, Siheung, Republic of Korea) was employed at 0.5 bar pressure and a rate of 1 m/min to achieve a 30% wet pick-up. UV irradiation was conducted by applying an energy of 24.6 J/cm^2^ to one side of the nylon fabric using a 360 nm LED lamp. The energy consumption during the photografting process was quantified using a WT500 Power Analyzer (Yokogawa, Tokyo, Japan) in conjunction with a PQ3198 Power Quality Analyzer (Hioki, Nagano, Japan). Subsequently, the fabricated filter underwent a washing procedure with distilled water to eliminate any residual, unfixed monomers, and initiator.

### 2.3. Characterization of the UV-Photografted Filters

Fourier-transform infrared spectroscopy (FT-IR), conducted with a Perkin Elmer instrument based in Waltham, MA, USA, was employed to verify the successful photografting of DMAPMAm onto the filter surface. Additionally, the fabricated textile filter underwent analysis using X-ray photoelectron spectroscopy (XPS) with monochromatic Al Kα radiation, utilizing the Theta probe system from Thermo Scientific (Kratos, Manchester, UK). Atomic ratios were derived from the XPS spectra, and the data were analyzed using Xpspeak41 software. For the observation of surface morphologies, field-emission scanning electron microscopy (FE-SEM) was utilized. The imaging was carried out with a Hitachi Co. Instrument, specifically the SU8010 model, located in Tokyo, Japan.

### 2.4. Evaluation of Dye Adsorption Abilities

Dye concentrations spanning from 25 to 500 mg/L were subjected to adsorption over a duration of 60 h using 25 to 100 mg of the fabricated filters. The assessment of dye adsorption capabilities was based on two criteria: dye removal efficiency and dye adsorption capacity.

The formula for calculating the dye removal efficiency is as follows in Equation (1).

(1) $$R(\%)=\frac{C_{0}-C_{t}}{C_{0}}\times 100$$

The dye adsorption capacity can be obtained by the following Equation (2).

(2) $$Qt=\frac{C_{0}-C_{t}}{m}V$$

where Q_t_ is the adsorption capacity at time t (mg/g) and R is the dye removal rate (%). C_0_ is the initial concentration of the dye (mg/L), C_t_ is the dye concentration at time t (mg/L), V is the volume of the dye solution (L), and m is the mass of the filter used (g).

For UV-Vis spectroscopy, a Shimadzu U2600 spectrophotometer (Shimadzu, Kyoto, Japan) was utilized. The assessment of dye adsorption capabilities was accomplished by quantifying the dye concentration, adsorption time, and initial concentration of the dye solution, and filter content both before and after the adsorption process.

Kinetic experiments were performed to determine the rate of adsorption reaction depending on adsorption time at room temperature with an initial dye concentration of 0.1 g/L. Subsequently, the remaining dye in the solution after adsorption was monitored over time, following the introduction of 25 mg of the filter photografted with 10 wt% DMAPMAm. The experiments were performed after controlling all variables. Kinetic experiments were analyzed using a pseudo-second-order Elovich kinetic model and intra-particle diffusion model. The pseudo-first-order and pseudo-second-order models are mathematical equations that elucidate the adsorption rate by tracking changes in the concentration of the adsorbent (i.e., the concentration of the dye solution) over time. The equations corresponding to the pseudo-second-order models for the adsorption process of the adsorbent in aqueous solution are presented in Equations (3)–(5), respectively.

(3) $$\frac{t}{q_{t}}=\frac{t}{q_{e}}+\frac{1}{k_{2}q_{e}^{2}}$$

(4) $$q_{t}=\frac{1}{\beta }\ln\left(\alpha \beta \right)+\frac{1}{\beta }\ln t$$

(5) $$q_{t}=k_{id}\times t^{1/2}+C$$

where q_e_ is the adsorbed amount per unit weight at equilibrium, t is the adsorption time, q_t_ is the adsorbed amount per unit weight after time t, k_1_, k_2_ and k_id_ are the rate constants, α is the chemical adsorption rate, and *β* is a coefficient related to the expansion of the covered surface, C is related to a boundary layer (mg/g).

For the thermodynamic experiments, the adsorption isotherms were determined by varying the initial dye concentration within the range of 25 to 500 mg/L. This variation aimed to explore the interaction between the UV-photopolymerized filter, acting as the adsorbent, and the dye, serving as the adsorbate. Subsequently, the Langmuir and Friedrich isotherm models were applied, and their outcomes were analyzed. The modified equations corresponding to these models are presented in Equations (6) and (7), respectively.

(6) $$\frac{1}{q_{e}}=\frac{1}{K_{L}}\frac{1}{a_{L}C_{e}}+\frac{1}{a_{L}}$$

where q_e_ is the adsorption amount per unit weight at equilibrium; K_L_ and a_L_ experimental constants related to the maximum adsorption capacity and adsorption rate, respectively; C_e_ is the concentration at equilibrium.

(7) $$\ln q_{e}=\ln K_{F}+\frac{1}{n}\ln C_{e}$$

where K_F_ is the experimental constant for the maximum adsorption capacity and $\frac{1}{n}$ is the separation factor. The Friedrich adsorption isotherm model is suitable for adsorption on polymeric layers, assuming that the heat of adsorption decreases exponentially with the difference in the extent of adsorbate coverage on the adsorbed surface.

## 3. Results and Discussion

### 3.1. Characterization of the Fabricated Filters

Nylon, chosen for its durability and its ability to undergo radical formation under UV irradiation, was selected as the substrate for the filter. To minimize steric hindrance, DMAPMAm was employed as a comonomer, and UV-A radiation was utilized to minimize damage to the fibers. Consequently, we fabricated UV-photografted filters using DETX as the photoinitiator. DETX possesses an absorption peak in the long-wavelength region, which aligns well with UV-A lamps.

**Figure 2:**
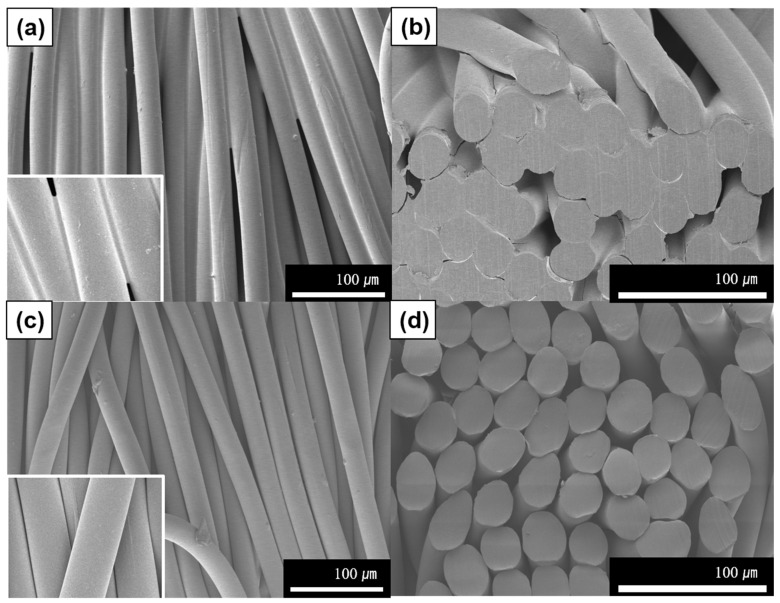
SEM micrographs of the UV-photografted filters: (**a**) surface, (**b**) side, (**c**) back, and (**d**) side of nylon substrate.

The SEM analysis of the surface of the UV-photografted filter was carried out to assess whether any residual modifications were present after the washing and drying process (refer to Figure 2). Since only one side of the nylon fabric underwent irradiation, we examined the UV-irradiated surface (Figure 2a), the cross-section (Figure 2b), the unirradiated back side (Figure 2c), and the cross-section of the nylon (Figure 2d). The SEM images reveal that the polymerization solution effectively infiltrated the spaces between the fibers on the surface. Moreover, the cross-section images indicate some minor penetration of the polymerization solution between the fibers. These observations collectively affirm that the surface underwent proper modification through UV photografting.

**Figure 3:**
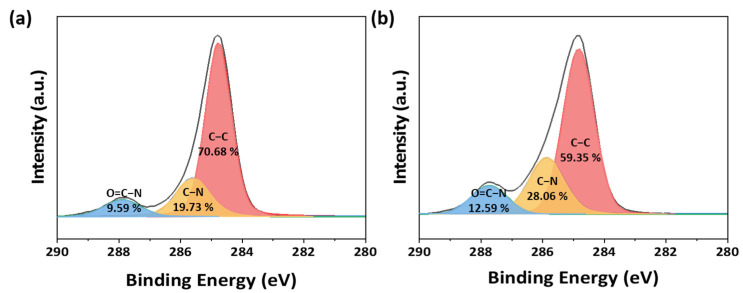
XPS C1s spectra of the nylon filter (**a**) before UV irradiation and (**b**) after UV irradiation.

FT-IR spectra of the Nylon substrate are in agreement with the polyamide-6 structure (Figure S1). The broad band at 3295 cm^−1^ appears, which is assigned to the N–H bending vibration of primary amine, and characteristic peaks assigned to C–H stretching of ethylene appear at 2935 cm^−1^ and 2862 cm^−1^. Moreover, the peak at 1632 cm^−1^ assigned to C=O amide I stretching and the peak at 1532 cm^−1^ assigned to N–H and C–N amide II stretching appeared [32]. However, DMAPMAm, a comonomer, also has an amide group and is characterized by C=O amide I stretching and N–H and C–N amide II stretching [33]. It is difficult to determine whether photografting occurs solely through FT-IR spectra. Therefore, to confirm that the DMAPMAm monomers were not merely superficially applied but rather covalently bound through UV irradiation onto the nylon substrate, we conducted a thorough examination of the filter’s surface chemical composition via XPS analysis. The multiplex spectra before and after UV irradiation for C1s in the range of 280–290 eV are illustrated in Figure 3. Observations from the spectra reveal a noticeable decrease in the C–C peak area, approximately from 70.68% to 59.35%, within the nylon filter after UV irradiation. Conversely, the C–N peak area and the ratio of O=C–N area increased. These changes indicate that DMAPMAm, featuring C–N and O=C–N bonds inherent in nylon, was covalently bonded to the radicals generated on the nylon surface by UV irradiation. Consequently, there was a marginal augmentation in the C–N and O=C–N peak intensities. Furthermore, the increase in the C–N and O=C–N bonds exceeded that of the C–C bonds, resulting in a reduction in the relative intensity of the C–C peak concerning the total peak. These outcomes strongly support the conclusion that UV irradiation facilitated the covalent bonding of the DMAPMAm monomer to the nylon substrate. Notably, since DMAPMAm grafted onto the fiber surface exhibits a pKa value of 8.831 [34,35], it is proficient in forming cations even under neutral conditions. Consequently, the final textile filter created possesses the ability to effectively bind anionic dyes that contain sulfonic acid groups found in dye wastewater. For a comprehensive understanding of the UV photografting of DMAPMAm and dye adsorption mechanisms, refer to Figure S2.

### 3.2. Dye Adsorption Abilities of Fabricated Filters

**Figure 4:**
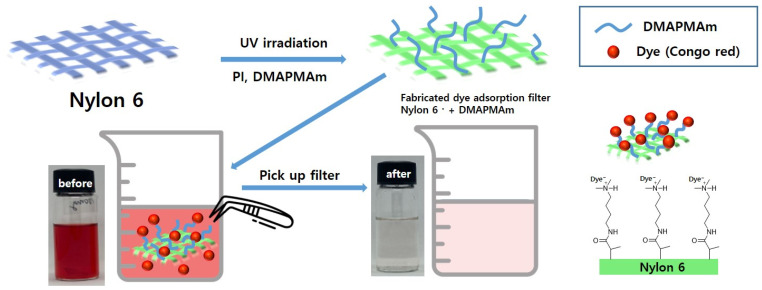
Schematic illustration of the process for dye adsorption of fabricated filters.

Adsorption of acid dye salts takes place through several mechanisms, including electrostatic attraction between the dye and the adsorbent, van der Waals forces, and hydrogen bonding, among others [34]. In the case of Congo Red, an acidic dye, the azo (–N=N–) and sulfur (–S–) groups within it can form hydrogen bonds with NH_2_ groups present in nylon, which serves as the base material for the UV-polymerized filter. Consequently, adsorption of Congo Red onto nylon is feasible. Additionally, the positively charged DMAPMAm is photografted onto the nylon surface, simplifying adsorption through electrostatic attraction with the negatively charged Congo Red dye. A schematic diagram illustrating the dye removal filter manufacturing process and the dye adsorption process is provided in Figure 4. Filters photografted with DMAPMAm, which has a pKa of about 8.8, react with hydrogen ions under pH conditions of 8.8 or lower, causing the nitrogen on the modified surface to become positively charged. Here, the negative charge of Congo Red in the dye solution and the positive charge of the surface nitrogen form ionic bonds. The dye is removed by picking it up from the dye solution using a filter combined with Congo Red.

**Figure 5:**
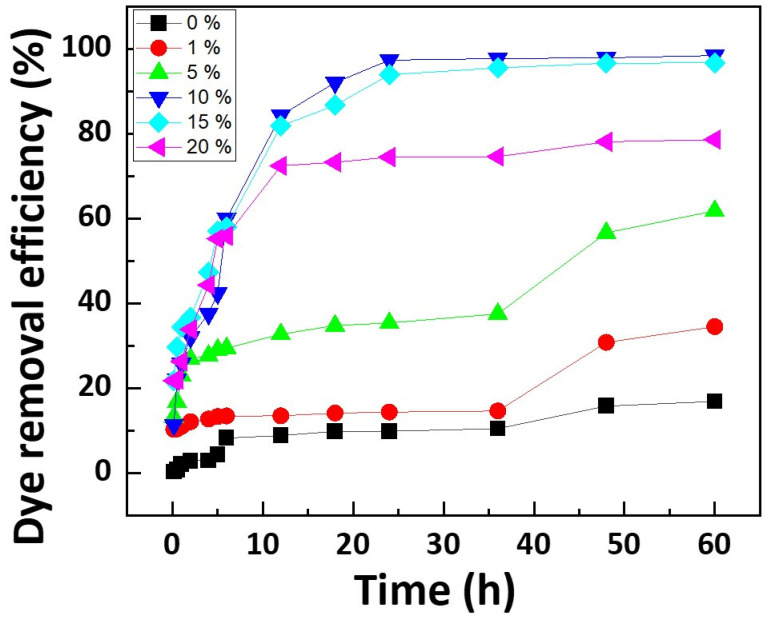
Dye removal efficiency according to DMAPMAm concentrations (initial concentration: 100 mg/L, dosage of filter: 25 mg).

Figure 5 illustrates the dye removal efficiency over time while varying the concentration of DMAPMAm. Here, 0% signifies nylon as the substrate without any added DMAPMAm. For the nylon filter photopolymerized with 10 wt% DMAPMAm, an impressive dye removal efficiency of 97.34% was achieved after 24 h. In contrast, the untreated nylon fabric, without DMAPMAm photografting, managed to remove only 16.86% of the dye after 60 h. Typically, nylon dyeing occurs at high temperatures of 90~100 °C, resulting in the untreated nylon substrate exhibiting low dye removal efficiency when the adsorption process takes place at room temperature. Moreover, since the adsorption experiment was conducted under neutral conditions, it is worth noting that the isoelectric point of nylon is 5.4 [36], giving it a limited affinity for anionic dyes. In contrast, DMAPMAm, with a pKa of 8.8 [35], exhibits a strong affinity for anionic dyes due to its abundant cations, even in a neutral state. Consequently, it can be deduced that the untreated nylon substrate is incapable of adsorbing dyes, and that UV photografting significantly enhances dye removal efficiency. Regardless of the DMAPMAm concentration, the removal efficiency showed a steady increase with time, accompanied by an increase in the amount of deposited dye. However, after 24 h, the degree of improvement in removal efficiency tended to decrease. Notably, with a DMAPMAm concentration of 10%, the dye removal efficiency rose with increasing concentration. Nevertheless, filters processed with a concentration of 15% or higher exhibited increased dye solution absorbance, attributable to residual elution of ungrafted DMAPMAm. Therefore, when assessing removal efficiency and adsorption capacity, it is appropriate to consider 24 h as a reference point. The optimal concentration of DMAPMAm, which demonstrates adsorption capacity on the photografted nylon filters, is 10 wt%. Consequently, in evaluating the dye adsorption capacity based on the initial concentration of the salt solution and filter content, a filter with a concentration of 10 wt% was utilized.

**Figure 6:**
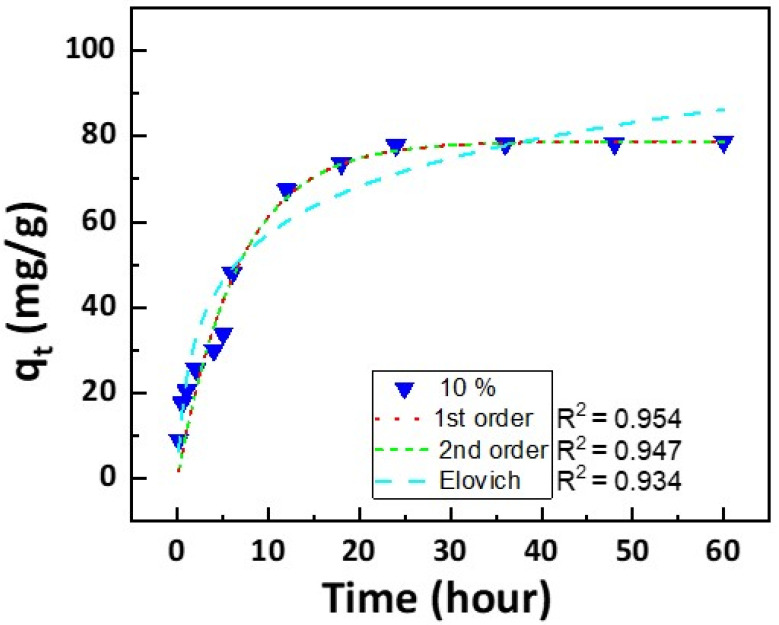
Regression analysis of the adsorption at 10% of DMAPMAm concentration.

**Figure 7:**
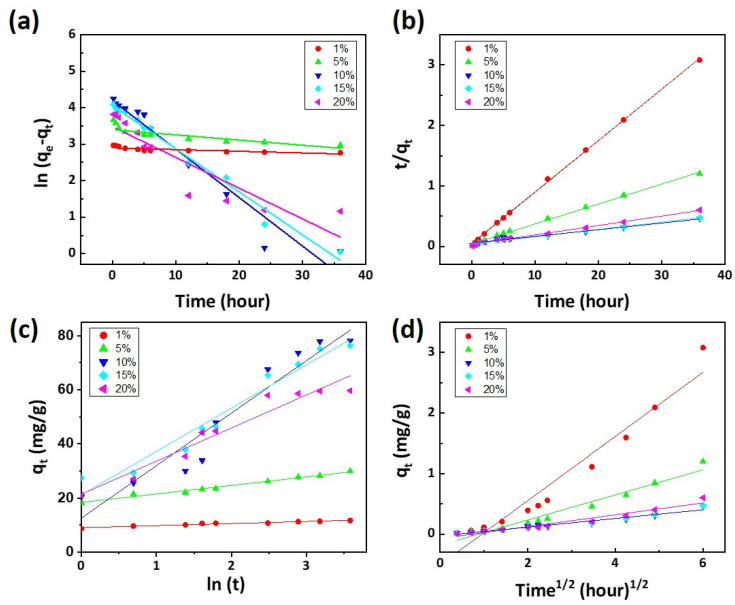
Regression analysis of the adsorption. (**a**) Pseudo-first-order model, (**b**) pseudo-second-order model, (**c**) Elovich equation, and (**d**) intra-particle diffusion model (initial concentration: 100 mg/L, dosage of filter: 25 mg).

Figure 6 illustrates a regression analysis of the correlation between the adsorption reaction rate and the processing concentration of the monomer DMAPMAm by applying the kinetic models (pseudo-first-order, pseudo-second-order, Elovich equation, and intra-particle diffusion model). First, Figure 6 shows the experimental data in the adsorption capacity of filters photografted with 10% of DMAPMAm over time, and the non-linear curves calculated from the three kinetic models. The pseudo-first-order model was found to be more suitable for expressing correlation than the pseudo-second-order model and Elovich equation. These results can be justified based on the correlation coefficients (R^2^), which were 0.954, 0.947, and 0.934, respectively. Figure 7a shows the regression results for the adsorption of filters under the pseudo-first-order model. The coefficients of correlation are within 0.667–0.983 (Table 2). Figure 7b shows the regression results for the adsorption of filters under the pseudo-second-order model. The coefficients of correlation are within 0.976–0.999 (Table 3). The pseudo-second-order model means that the reaction rate of a substance is proportional to the square of the concentration of the substance. Figure S3a illustrates the adsorption capacity of the photografted filter in relation to the monomer concentration, and Figure S3b shows the progression of dye adsorption over time. In Figure S3, the experimental q_e_ value stands at 78.73 mg/g. Notably, the pseudo-second-order model’s q_e_ value is 89.82 mg/g (Table 3). The experimental data, q_e(exp)_ and q_e(cal)_, calculated by the equation have similar tendencies. When the monomer concentration exceeds 10%, the adsorption capacity and the coefficient k_2_, which represents the adsorption amount per unit time, tend to rapidly decrease. For all DMAPMAm concentrations, the pseudo-first-order model is suitable for showing the correlation with reaction speed, and at 10% concentration, the optimal conditions, the pseudo-second-order model is suitable. Figure 7c shows the regression results for the adsorption of filters using the Elovich equation. It is widely used as an equation representing chemical adsorption between solids and liquids. The coefficient α is related to the chemical adsorption rate and tends to increase as the monomer concentration increases. β is related to the expansion of the screen of the covered surface, so above a certain concentration, it is already adsorbed as dye molecules and shows a low value. As the concentration of DMAPMAm increases, the α value increases, so the initial adsorption rate is fast. As the concentration increases, the β value decreases, resulting in less desorption. At concentrations of 15% or more, the β value increases and desorption increases (Table 4). From the R^2^ value in the range of 0.961–0.984, it can be inferred that the Elovich model can be accepted as one of the characteristics of the adsorption reaction. Figure 7d shows the regression results for the adsorption of filters under the intra-particle diffusion model. The filter adsorption breakpoints for Congo Red are 6 and 18 h. The first rapid step is due to the diffusion of Congo Red through the solution to the surface of the filter, while the second step represents the diffusion of Congo Red into the internal pores of the filter. The third step is the final equilibrium [37]. The *k*_id_ value decreases after the first breakpoint, and decreases significantly again after the second breakpoint, indicating that equilibrium is reached and the adsorption rate slows down. The C value, which represents the boundary layer, increased in the second step, but increased slightly in the third step as it reached equilibrium. Since the correlation coefficient R^2^ is within the range of 0.972–0.984, it can be said to be a suitable model to represent the adsorption rate of the DMAPMAm photografted filter for Congo Red (Table 5).

### 3.3. Dye Adsorption Abilities According to Initial Concentration of Dye Solution

**Figure 8:**
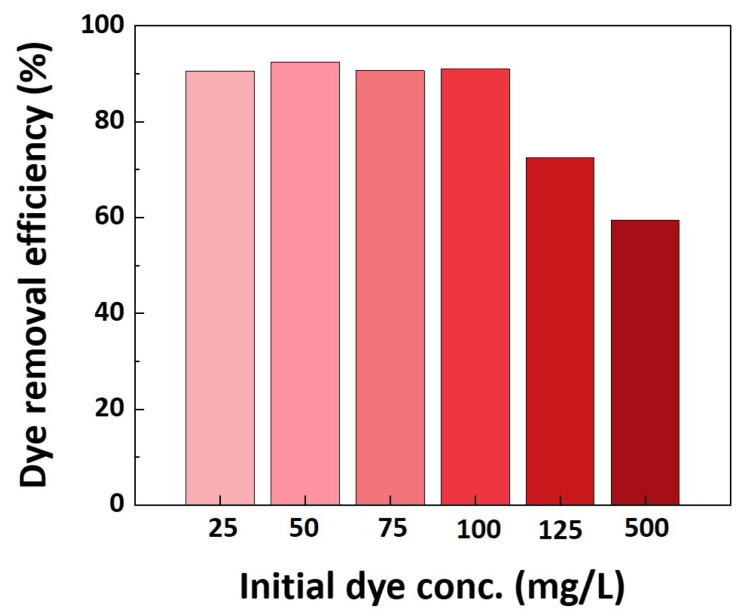
Dye removal efficiency of fabricated filters according to concentration of initial dye solution (monomer concentration: 10%, dosage of filter: 25 mg).

Figure 8 illustrates the dye adsorption capacity concerning the initial dye solution concentration. When concentrations fall below 100 mg/L, the dye removal efficiency exceeds 90%. This phenomenon results from the ionic bonding occurring between the protonated amine group of the photografted filter and the acidic dye. Even as the concentration increases below 100 mg/L, the dye adsorption capacity remains unchanged due to the presence of functional groups that can react with the filter. However, as concentrations exceed 125 mg/L, both the dye removal efficiency and dye adsorption capacity experience a significant decline. This decline can be attributed to the absence of positively charged functional groups on the filter surface, which are required to react with the dye. Nevertheless, it is important to note that dyeing processes typically operate at concentrations of 5% or lower on the fiber. Consequently, the concentration of dyeing wastewater generally remains at or below 100 mg/L. Consequently, the filter exhibits ample adsorption properties for the removal of residual dyes from dyeing wastewater.

The Langmuir adsorption isotherm model is well-suited for adsorption onto monolayers with uniform surfaces. According to the Langmuir model, when a filter is manufactured with a 10% monomer concentration and adsorbed at 25 mg, the theoretical maximum adsorption capacity expressed in a_L_ is 86.96 mg/g, and the correlation coefficient R^2^ is 0.96. This means that up to 86.96 mg/g of Congo Red can be adsorbed as a uniform monolayer on the manufactured filter, which is similar to the experimental data of the adsorption capacity in equilibrium under the same conditions (Table 6). On the other hand, the Friedrich adsorption isotherm model is appropriate for adsorption on multimolecular layers, operating on the assumption that the heat of adsorption diminishes exponentially with variations in the degree of adsorbate coverage on the adsorbed surface. In the Friedrich model, the N value is related to adsorption preference and adsorption capacity, and when it is greater than 1, it means that adsorption is f. Therefore, since the N value is 2.38, it can be said that the filter adsorbs Congo Red favorably (Table 6). Additionally, both the Langmuir and Friedrich models yielded R^2^ values of 0.96 and 0.88, respectively. These results imply that the filter’s surface was uniformly photografted and adsorbed as a monolayer. Therefore, based on these findings, it can be concluded that the Langmuir model is better suited to represent the adsorption interactions of the fabricated filter.

### 3.4. Dye Adsorption Abilities of the Fabricated Filters by Dosage

**Figure 9:**
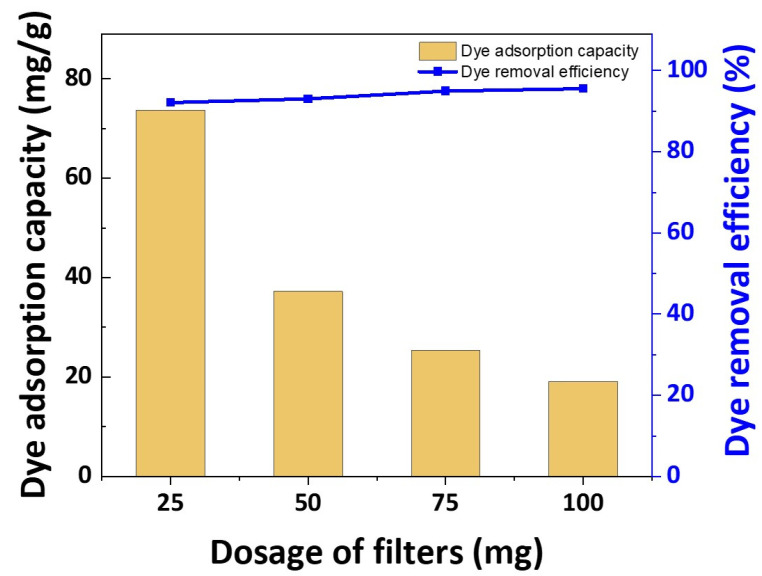
Dye adsorption abilities according to dosage of filters (initial concentration: 100 mg/L, monomer concentration: 10%).

Figure 9 illustrates the relationship between dye removal efficiency and dye adsorption capacity as the filter content increases. Notably, the incremental increase in filter content exerts minimal influence on dye removal efficiency, which already exceeds 90%, even when only 25 mg of filter are introduced into the brine solution. Furthermore, in Figure 9, it is evident that, following a 24 h period, the adsorption capacity of the untreated nylon substrate stood at 0.21 mg/g, whereas that of the fabricated filter at 10% concentration was 77.88 mg/g, while the fabricated filter at a 10% concentration demonstrated an impressive adsorption capacity of 77.88 mg/g. This remarkable result signifies that the photografted filter can adsorb approximately 77.88 milligrams of dye per gram, a figure that is 370 times higher than that of the untreated nylon substrate.

Meanwhile, in the dye adsorption capacity in Figure 9, it appears that as the filter content increases, the amount of adsorption per gram decreases. It does not mean that adsorption capacity of the filters decreases. It means that sufficient adsorption is possible with only 25 mg of the filters in the 100 mg/L dye solution, meaning that a surplus filter exists. In other research on adsorption of acid dyes, more than 0.1 g of absorbent was used, or another treatment was conducted to achieve 90% dye removal efficiency [38,39,40,41,42,43,44,45] (Table 7). Therefore, if a UV-photografted filter is used for dye adsorption in wastewater, it is more economical and has an excellent effect in dye removal efficiency even if only using 25 mg of filter without any treatment process.

## 4. Conclusions

In this study, we created dye adsorption filters capable of adsorbing dyes in salt solutions through the surface modification of nylon fabric using UV irradiation. This method, which is simpler and more effective than thermal or catalytic polymerization, was employed to demonstrate the adsorption performance of these filters. The surface of the filter, modified with DMAPMAm, exhibited a positive charge, featuring amine and imine groups, as confirmed by XPS analysis. When the polymerized filter was immersed in a 0.1 g/L salt solution for 24 h, the dye removal efficiency exceeded 98%, allowing the removal of 77.88 mg of dye per gram of filter. This demonstrates the remarkable adsorption capacity, enabling the removal of dyes with a minimal amount of filter material. Additionally, since the process does not generate sludge post-dye adsorption, the dyes can be efficiently removed by merely extracting the filter without requiring a sludge separation device. This streamlined approach simplifies dye removal and reduces the energy consumption typically associated with adsorbent removal. It is worth noting that optimizing the adsorption capacity can be achieved by controlling other variables related to adsorption efficiency, such as temperature, or modifying processing conditions. In summary, the dye adsorption filter presented in this study offers an eco-friendly solution, manufactured using UV irradiation, with exceptional dye adsorption capacity. It has the potential to address the issue of dyeing wastewater disposal in the dyeing industry, offering a more straightforward and environmentally friendly approach.

## Figures and Tables

**Table 1:** Characteristics of substrate fabric.

Composition	Weave	Yarn	Yarn Count	Density	Mass/Unit Area (g/m^2^)
Nylon 6 100%	Plain	500 D/96 F	59	51 × 38	205

**Table 2:** Parameters and correlation coefficients obtained from the analysis of adsorption kinetics under pseudo-first-order model.

Concentration (%)	Parameter			
	q_e(exp)_ (mg g^−1^)	q_e(cal)_ (mg g^−1^)	k_2_ (g mg^−1^ h^−1^)	R^2^
1	27.59	13.91	1.568	0.667
5	49.44	32.82	0.441	0785
10	78.73	60.01	0.281	0.943
15	77.34	74.66	0.205	0.983
20	62.86	78.79	0.150	0.816

**Table 3:** Parameters and correlation coefficients obtained from the analysis of adsorption kinetics under pseudo-second-order model.

Concentration (%)	Parameter			
	q_e(exp)_ (mg g^−1^)	q_e(cal)_ (mg g^−1^)	k_2_ (g mg^−1^ h^−1^)	R^2^
1	27.59	17.08	0.008	0.999
5	49.44	36.28	0.016	0.998
10	78.73	89.82	0.990	0.997
15	77.34	81.71	0.004	0.991
20	62.86	64.76	0.007	0.973

**Table 4:** Parameters and correlation coefficients obtained from the analysis of adsorption kinetics using Elovich equation.

Concentration (%)	Parameter		
	α (mg g^−1^ h^−1^)	*β*	R^2^
1	67.91	0.408	0.974
5	89.00	0.175	0.984
10	86.05	0.055	0.961
15	88.13	0.073	0.978
20	116.24	0.098	0.971

**Table 5:** Parameters and correlation coefficients obtained from the analysis of adsorption kinetics under intra-particle diffusion model at 10% DMAPMAm concentration.

Step	Parameter		
	C (mg g^−1^)	*k_id_* (mg g^−1^ h^−0.5^)	R^2^
I	0.00	19.67	0.988
II	25.64	7.327	-
III	30.60	0.765	0.960

**Table 6:** Isotherm parameter of fabricated filters for adsorption of Congo Red.

Isotherm Model	Langmuir			Freundlich		
Parameter	K_L_	a_L_ (mg g^−1^)	R^2^	K_F_ (L mg^−1^)	N	R^2^
	0.067	86.96	0.96	0.013	2.38	0.88

**Table 7:** Dye adsorption capacities of other adsorbents.

Absorbents	Weight (mg)	Dye Conc. (mg L^−1^)	Adsorption Capacity (mg g^−1^)	Treatment
Biochar [38]	30	500	210	Stirring
NiFeLDH/Au [39]	10	10	60	pH 4.8
LDH [40]	30	100	33	pH 4
Activated carbon [41]	100	100	76	stirring
NiFe/carbon [42]	20	50	291	stirring
apricot stone [43]	100	100	20	stirring

## Data Availability

Data are contained within the article.

## Supplementary Materials

The following supporting information can be downloaded at: https://www.mdpi.com/article/10.3390/polym16010015/s1, Figure S1: FT-IR spectra of the Nylon substrate and UV-photografted filter.; Figure S2: UV photografting of DMAPMAm and dye adsorption mechanism. Figure S3: Dye adsorption capacity according to (a) concentration of DMAPMAm (b) over time.
