# Supplementary material for: Highly Sustainable Dyes Adsorption in Wastewater Using Textile Filters Fabricated by UV Irradiation
Source: Polymers (Basel). 2023 Dec 19;16(1):15. doi: 10.3390/polym16010015 (PMC10780358; doi:10.3390/polym16010015)
Supplement: Supplementary file 1 [file polymers-16-00015-s001.zip › polymers-2683947-supplementary.pdf]

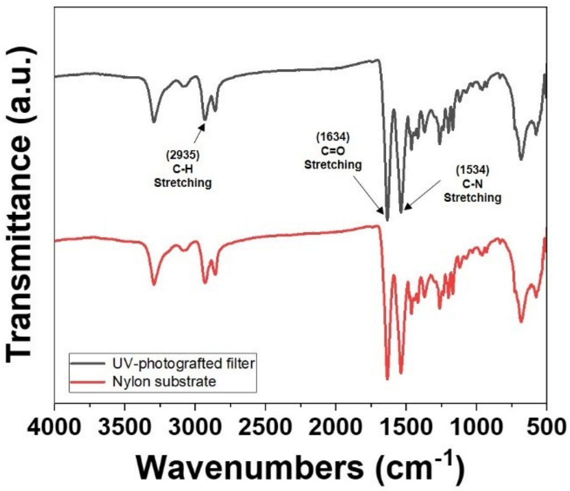

Figure S1: FT-IR spectra of the Nylon substrate and UV-photografted filter.

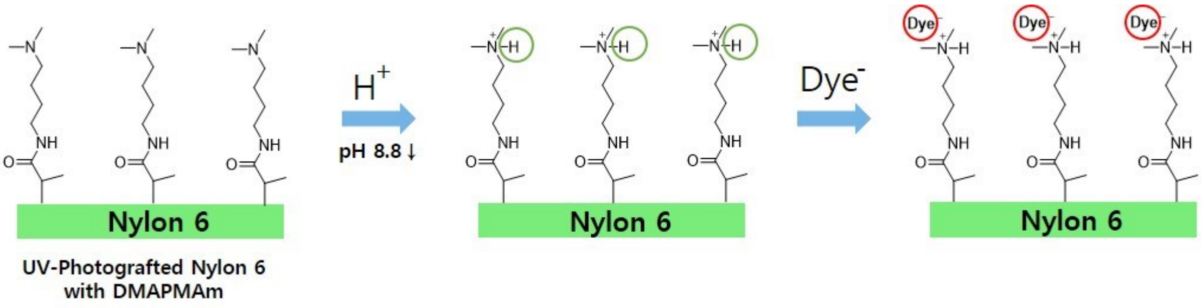

Figure S2: UV-photografting of DMAPMAm and dye adsorption mechanism.

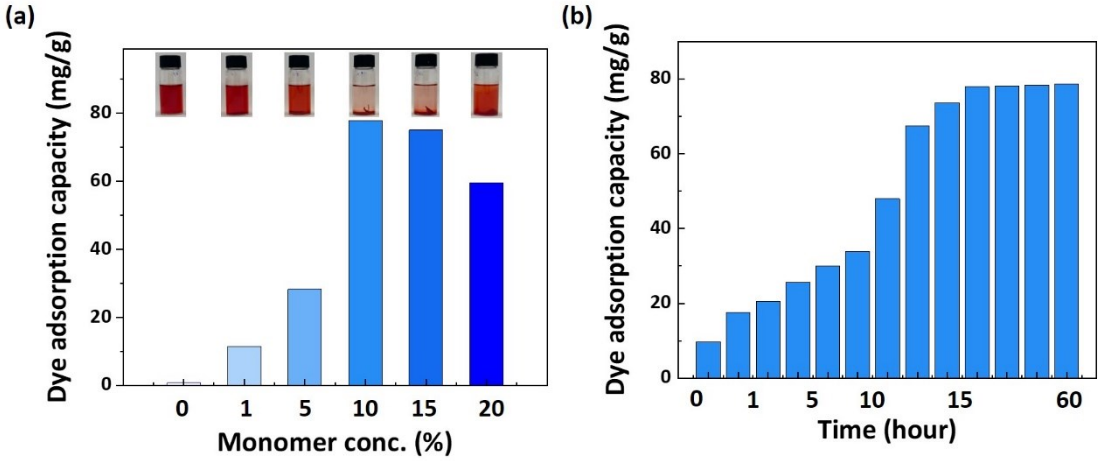

Figure S3: Dye adsorption capacity according to (a) concentration of DMAPMAm (b) over time.
